# Oxford at Its Best

**Published:** 1892-12-24

**Authors:** 


### OXFORD AT ITS BEST.

(*By an Occasional Correspondent.*)

You have been severe upon certain faults which pervade Oxford society and life, as shown by its indifference to the well-being of the Radcliffe Infirmary. May I crave a short space to show a better and more worthy side of the life of this great university city?

On Tuesday, Miss Edith May, one of the daughters of the Reverend Professor Pritchard, was married at St. Giles' Church, Oxford, to Professor J. Bretland Farmer, of the Royal College of Science, South Kensington. The Bishop of Worcester officiated, assisted by his Chaplain and the Rector of St. Giles. The church was crowded, and it was pleasant to notice the large number of poor people who were present. Professor Farmer is a Fellow of Magdalen College, and the Magdalen College choir took part in the service, which was fully choral. The bride was magnificently attired, and Master Simeon, who acted as train-bearer, although but seven years old, performed his task with a gravity and grace which could not have been exceeded by the most experienced courtier. It would be out of place in your columns to attempt to describe the dresses, many of which were charming, and especially one worn by a sister of the bride. The service opened with a beautiful hymn, commencing "Oh, perfect love," based upon the words, "The Lord do so to me and more also, if aught but death part thee and me." The rendering of this hymn by the Magdalen Choir was most touchingly effective, and the beautiful words found an echo in the hearts of many hearers. Professor Pritchard, although eighty-three years of age, was present, and gave the bride away. It was pleasant to see the joy which he experienced in being able to undertake this pleasant duty, and its discharge seemed to rejuvenate him wonderfully. There were some wondrous bouquets, dedicated apparently to Christmas, as the bridesmaids' consisted of holly and red berries, and one of the guests had an extensive arrangement of mistletoe and chrysanthemums. The Bishop of Worcester, who is Professor Pritchard's oldest friend, performed the service in a most fatherly and benign manner. It was touching to see how gently, but firmly, he instructed the bridegroom as to his duties step by step.

After the service the wedding party adjourned to Professor Pritchard's house, where the presents were exhibited. These presents, some 150 in number, included a very handsome set of silver candlesticks from the Fellows of Magdalen College, more than one silver tea service, a complete set of silver for the table, silver dishes, claret jugs, salvers, a silver-mounted dressing-case, and many other beautiful and valuable objects. The gem of the collection was a grandfather's clock in delf, which won all hearts. There were many examples of old and modern china, including a splendid French china dinner service of an ancient pattern, which must have been most valuable. Diamond rings, crescents, and a magnificent garnet necklace and other ornaments were much admired; but the real charm of these presents consisted in the evident care with which they had been chosen, and although, taken collectively, they represented much that was admirable, each individual present had a special character all its own, which proved that its selection had been a labour of love.

Now, it will be asked, why do I claim that this marriage shows Oxford society in a better light. Because all Oxford was represented, not only at the ceremony and amidst the presents, but the proceedings produced an abundance of evidence of the hearty and cordial interest which this marriage excited amongst all classes. There seemed to be a genuine desire to show that the bride and bridegroom's efforts in the past, not only to make themselves agreeable and helpful to others in their own station, but to minister in a quiet but effective way to the needs of the poor, were held in grateful remembrance. We Oxford people are not a wealthy community, but we are distinctly sociable and kind-hearted, and I venture to think that the wedding at St. Giles' on Tuesday, and every incident in connection with it, spoke eloquently in testimony of the soundness of the spirit which animates Oxford society at the bottom. We tried to give the bride and bridegroom full assurance of our genuine sympathy with their joy and happiness, and to express our belief that the tender charity and steadfast faith of the bride, which everyone of us has learned to honour, had won for her the joy which calms all earthly strife.

